# Chemical Structures of Adhesive and Interphase Parts in Sucrose/Citric Acid Type Adhesive Wood-Based Molding Derived from Japanese Cedar (*Cryptomeria japonica*)

**DOI:** 10.3390/polym13234224

**Published:** 2021-12-02

**Authors:** Daisuke Ando, Kenji Umemura

**Affiliations:** 1 Institute of Wood Technology, Akita Prefectural University, 11-1 Aza Kaieizaka, Noshiro 016-0876, Akita, Japan; 2 Research Institute for Sustainable Humanosphere, Kyoto University, Gokasho, Uji 611-0011, Kyoto, Japan; umemura@rish.kyoto-u.ac.jp

**Keywords:** chemical reaction mechanism, citric acid, 5-HMF, HSQC-NMR, sucrose, wood adhesive, wood-based molding

## Abstract

In sucrose/citric acid based wood adhesive, the detailed bonding mechanism has still been unknown. Here, we investigated the detailed chemical structures of this adhesive wood (Japanese cedar)-based molding by using heteronuclear single quantum coherence–nuclear magnetic resonance (HSQC-NMR). NMR peaks associated with the furan-type structure appeared, suggesting that the furan compound was formed from sucrose and converted to a furan polymer during the adhesive process and that some of the furan structures in the polymers were ester-bonded with citric acid. The secondary forces between the furan polymers and wood components were thought to contribute to the adhesive effect. In our analysis of the interphase structure, primary hydroxyl groups of both polysaccharides and of lignin substructures were found to be esterified with citric acid. Additionally, some of the glycosidic bonds in polysaccharides were cleaved during the acidic condition produced by citric acid. The above results provided evidence of the polymerization of sucrose-derived 5-HMF, the esterification of wood components, and the degradation of polysaccharides during the molding process. Citric acid functioned as a clamp between the obtained furan polymer and the wood components. The sucrose/citric acid based wood adhesive can be defined as a hybrid-type wood adhesive, involving both secondary forces and chemical bonding interactions.

## 1. Introduction

Wood biomass has long been used as a structural material. In many cases, wood adhesives derived from petrochemicals (phenol-formaldehyde adhesives, urea-formaldehyde adhesives, melamine-formaldehyde adhesives, phenol-resorcinol-formaldehyde adhesives and polymeric 4,4′-diphenyl methane diisocyanate (pMDI) adhesives) have been essential for the utilization of wood biomass [1,2,3]. However, in recent years, our social obligation to mitigate environmental problems such as global warming and environmental pollution has led to a global imperative to reduce consumption of petroleum products. Novel bio-based wood adhesives, such as those based on proteins [4,5], oils [6,7,8,9], carbohydrates [10], tannin [11,12], lignin [12], and citric acid [13,14,15] have received a lot of attention as potential alternatives. Our group has focused on sugar-based wood adhesives.

In the past, carbohydrates, including various sugars (glucose, sucrose, etc.) and polysaccharides (wheat flour, starch, etc.), have been examined as wood adhesives [16]. The carbohydrates are assumed to functionalize as co-reactants with phenolic-type [17,18] and other resins [19,20] or as adhesives in their own right [21,22]. In some cases, carbohydrates, especially low-molecular-weight compounds such as monosaccharides and oligosaccharides, have been dehydrated under acidic conditions to produce 5-hydroxymethyl-2-furfural (5-HMF) and other furan compounds [23]. Those compounds have been considered to react to resins or themselves function as adhesives [21]. Moreover, in the case of the reaction of sugars, the subsequent decomposition of 5-HMF can proceed to produce levulinic acid and formic acid under such conditions [24,25]. Therefore, in carbohydrate-type wood adhesive, these organic acids, produced depending on the reaction conditions, may then polymerize by themselves or with furans, and bind to wood.

Recently, sucrose/citric acid type adhesives have been reported [26,27,28,29]. The optimum conditions depend on the wood particle sizes. In the case of plywood, a curing pretreatment of the sucrose and citric acid mixture is required. Sun et al. [30] and Zhao et al. [31] reported an analysis of this pretreated adhesive using FT-IR, HPLC, Py-GC/MS, and ^13^C-NMR. In their research, 5-HMF and other furan compounds were detected during the curing process. Then, it was assumed that those compounds reacted with citric acid to form insoluble material. However, there remain some unknown details about the adhesive chemical structures in the moldings. Thus, we attempted to obtain more detailed information about the sucrose/citric acid adhesive, and especially to determine the adhesive type, i.e., secondary forces vs. chemical bonding, using a non-degradative analysis, gel-state 2D (HSQC-) NMR.

In a previous report [32], the structure of the citric acid-based adhesive in the molding was investigated with HSQC-NMR analysis to clarify the esterification of wood components with citric acid. It is elucidated that the citric acid type adhesive is a chemical covalent-bonding-type adhesive and citric acid functions as a clamp between wood powder particles. On the other hand, in addition to esterification, it was reported that its acidity promoted the cleavage of glycosidic linkages to decompose polysaccharides, too.

In the sucrose/citric acid type adhesive process, which is more complicated than the citric acid type one, multi reactions of sucrose, citric acid, and wood components are expected to proceed. In this study, we focused on this adhesive and tried to elucidate the reactions taking place during the adhesive process, using HSQC-NMR analysis to clarify the adhesive mechanism.

## 2. Materials and Methods

### 2.1. Materials

The wood powder was derived from Japanese cedar (*Cryptomeria Japonica*). Sucrose (Guaranteed Reagent) and citric acid (Extra Pure Reagent) were purchased from Nacalai Tesque, Inc. (Kyoto, Japan) and were ground with a mortar in order to obtain a powder with a particle size less than 250 μm by using a sieve of 250 μm mesh size. These materials were dried in vacuo at 60 °C for 15 h. DMSO-d_6_ and pyridine-d_5_ were purchased from WAKENYAKU CO., LTD. (Kyoto, Japan).

### 2.2. Molding Preparation

The wood powder (6.4 g), the sucrose powder (1.2 g) and the citric acid powder (0.4 g) were mixed until the mixture was uniformly distributed. The sucrose and citric acid content (wt%) together made up 20% of the substrate. A cylindrical mold with a 70 mm inner diameter was used for the moldings. The powder mixture produced as described above was poured into the molds and hot-pressed at 200 °C and 4 MPa for 10 min. The obtained moldings were stored in a desiccator with silica gel.

### 2.3. Sample Preparation in HSQC-NMR

Ball-milled NMR samples were prepared according to the method previously described [33]. The crushed molding (200 mg) was ball-milled with a planetary Mono Mill PULVERISETTE 6 classic line (Fritsch GmbH, Idar-Oberstein, Germany), with vibration at 600 rpm in zirconium dioxide (ZrO_2_) vessels (12 mL) containing ZrO_2_ 50 balls (5 mm × 5 mm). The grinding time to obtain samples was 80 min, in 20 min grinding/10 min intervals for four cycles to avoid excessive heating.

### 2.4. 2D HSQC-NMR Spectroscopy

**Figure 1:**
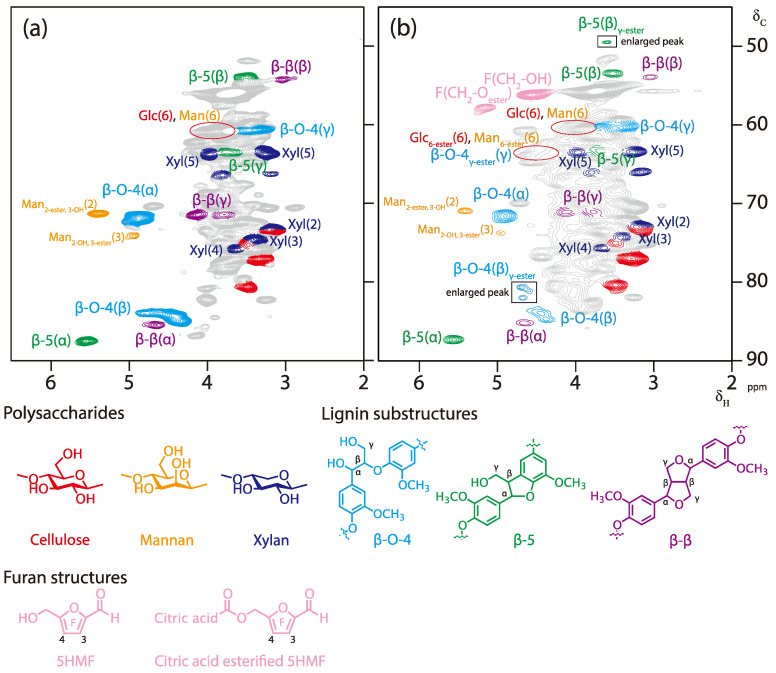
HSQC-NMR spectra in the aliphatic region of wood (Japanese cedar) (**a**) and the sucrose/citric acid type adhesive wood-based molding (W80) (**b**).

**Figure 2:**
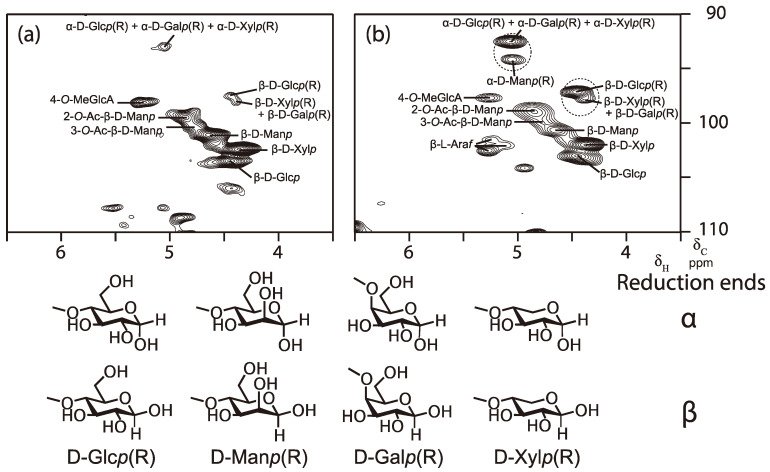
HSQC-NMR spectra in the anomeric region of the wood (Japanese cedar) (**a**) and the sucrose/citric acid type adhesive wood-based molding (W80) (**b**).

**Figure 3:**
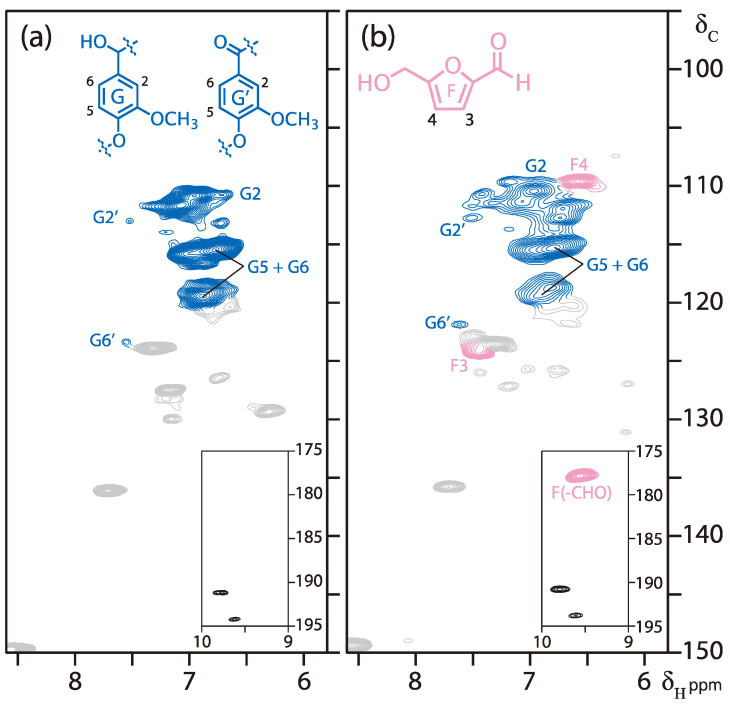
HSQC-NMR spectra in the aromatic region of the wood (Japanese cedar) (**a**) and the sucrose/citric acid type adhesive wood-based molding (W80) (**b**).

A Bruker Avance 800 MHz spectrometer (Bruker Biospin, Bruker, Billerica, MA, USA) at 323 K was used. The central DMSO solvent peak was used as the internal standard (δ_H_ 2.49, δ_C_ 39.5 ppm). An HSQC experiment was applied to the ^1^H−^13^C correlation experiment (Bruker standard pulse sequence “hsqcetgpsp.3”). The solvent was premixed with DMSO-d_6_/pyridine-d_5_ (*v*/*v*, 4/1), and the samples were allowed to swell in the solvent. The spectral widths were 7788 and 21,135 Hz for the ^1^H and ^13^C dimensions, respectively. The number of collected complex points was 1536 for the ^1^H dimension, with a recycle delay of 1.5 or 2.5 s. The number of transients was 16 or 32, and 256 time increments were always recorded in the ^13^C dimension. Gaussian multiplication (LB = −1.00) and squared sine bell (SSB = 2) window functions were applied in the ^1^H and ^13^C dimensions, respectively. Prior to the FT, the data matrices were zero-filled up to 2048 and 1024 points in the ^1^H and ^13^C dimensions, respectively. The spectra of wood powder, shown in Figure 1a, Figure 2a and Figure 3a), were referred from those in the previous report [32] for comparing with the molding, because the same wood powder is the starting material in this study.

## 3. Results and Discussion

In general, there are two different reaction fields (the adhesive and the interphase parts) in the adhesive process. In the adhesive part, the reaction of the adhesive with itself (in this case, sucrose and citric acid) proceeds. In the interphase part, wood is involved in the reaction in addition to the adhesive compounds. We analyzed the sucrose/citric acid type adhesive wood-based molding with HSQC-NMR spectroscopy and considered adhesive and interphase regions in the molding separately to obtain a comprehensive understanding of the structures of the molding.

The obtained HSQC-NMR spectra of the wood powder (Japanese cedar) and the molding were assigned as in the previous report (Figure 1). In the spectrum of the wood powder and the molding (Figure 1), the basic signals of polysaccharides (cellulose (red), glucomannan (orange), xylan (dark blue)) and lignin (β-O-4 (cyan), β-5 (green), β-β (purple)) substructures were confirmed by comparison with those in the literature [33,34,35,36,37].

### 3.1. Adhesive Structure of the Molding: Formation of Furan Structures

In prior studies [30,31], various furan compounds have been detected as being present in the extraction and the degradation processes of the sucrose/citric acid type adhesives. Sucrose is known to be dehydrated to furan under high-temperature and high-pressure conditions [38,39,40]. We focused on the adhesive’s chemical structure change in the molding with HSQC-NMR analysis.

First, we focused on the anomeric regions of 3.5–6.5/90–110 ppm (Figure 2). In the molding’s spectrum, an anomeric signal in the glucose unit of sucrose at 5.35/91.6 ppm was not detected, but reduction end (α and β) signals at 5.06/92.6 and 4.45/97.2 ppm increased. Therefore, the glycosidic linkage between the glucose and fructose in sucrose was completely cleaved under the adhesive condition (200 °C, 4 MPa, 10 min).

Next, we focused on the aromatic regions (Figure 3) of 5.8–8.6/95–150 ppm. Not only the signals of lignin’s aromatic ring (blue) but also those of the furan ring (pink) in 5-HMF (6.58/109.7 ppm and 7.45/124.2 ppm) were observed. The aldehyde signal of 5-HMF (9.55/177.8 ppm) was also observed. Likewise, the signal of the hydroxyl methyl in 5-HMF was observed at 4.54/56.2 ppm on the aliphatic regions (Figure 1b) of 2.0–6.5/45–90 ppm. This confirmed that the thermal degradation of sucrose proceeded under the citric acid condition to produce 5-HMF or a 5-HMF-type structure. Moreover, the methyl signal of the esterified 5-HMF was detected at 5.15/57.8 ppm. Therefore, citric acid was shown to form an ester linkage to 5-HMF, as in mumefural [41]. In other words, citric acid functioned not only as an organic acid but also as a reactant.

**Scheme 1:**
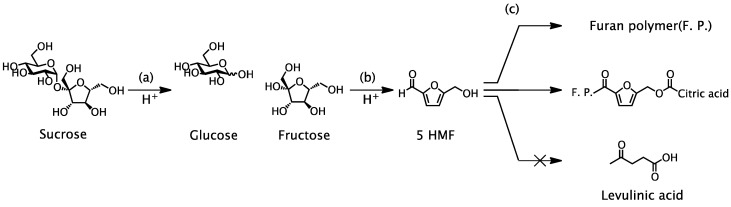
Structure change of sucrose under the adhesive process. (**a**) Degradation of sucrose, (**b**) conversion to 5-HMF, (**c**) conversion of 5-HMF.

Signals of levulinic acid (2.50/27.6, 2.07/29.3, and 2.69/37.4 ppm) were observed slightly in the alkyl region (0–4/0–50 ppm). Other organic acids, such as lactic acid, etc., that are known to be produced under sucrose thermal degradation, were barely present. Namely, cleavage of the glycosidic linkage and the production of furan structure were the main reactions under this adhesive condition, although subsequent conversion to organic acids such as levulinic acid occurred in trace amounts (Scheme 1).

These results clarified that the polymer linking furans and citric acid was predominant in the adhesive part and that the citric acid was linked to furans or to furan polymers. In other words, the adhesive functioned as a polymer during the molding process.

### 3.2. Interphase Structures between Adhesive and Wood: Esterification and Polysaccharide Degradation

In the interphase region, the prime concern is whether or not a chemical bond between the wood and adhesive is formed. In this session, we tried to elucidate the interphase structures between the adhesive and wood with HSQC-NMR spectroscopy: namely, whether change could be detected in the wood component structures as a result of the adhesive process.

In the spectrum of the molding (Figure 1b), some esterified signals were observed, as in the citric acid type wood-based molding. In the molding’s aliphatic region (2.0–6.5/45–90 ppm), overlapping signals appeared at approximately 4.18/64.5 ppm (red circle), which were namely the esterified 6-position signals of polysaccharides (such as cellulose and galactoglucomannan) and the H_γ_/C_γ_ signal of the γ-esterified β-O-4 lignin substructure.

Next, the H_β_/C_β_ signals of β-O-4 and β-5 lignin substructures were searched for. Indeed, H_β_/C_β_ signals of γ-esterified β-O-4 and β-5 lignin substructures were found at 4.61/81.0 (cyan) and 4.72/82.1 ppm (cyan) and at 3.60/49.5 ppm (green) when the regions in the molding’s spectrum were enlarged. This result clarified that a small amount of those lignin structures was esterified.

**Figure 4:**
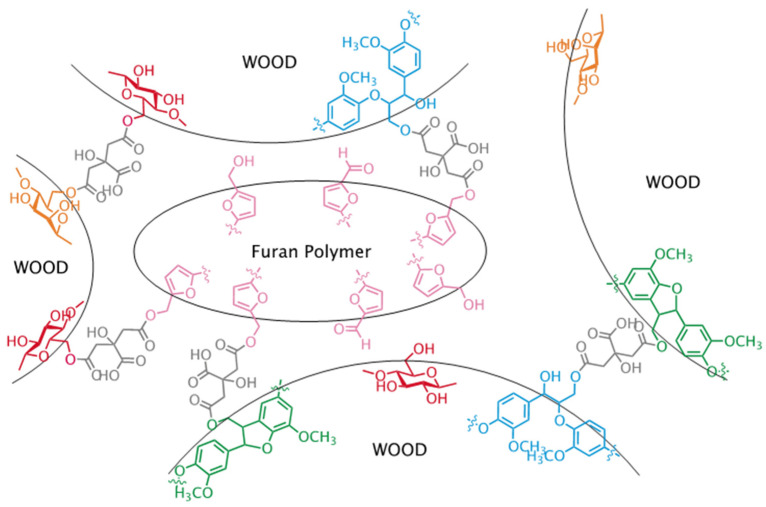
Structures between wood powders.

Some of the wood components were esterified with citric acid although the content of the citric acid in the sucrose/citric acid type adhesive wood-based molding was lower than that in the citric acid type adhesive wood-based molding [32]. Namely, it was elucidated that the interface between the furan polymer and wood powder was bonded via citric acid (Figure 4). Thus, citric acid was involved in not only the thermal conversion of sucrose to furan but also in esterification.

Next, in the anomeric region (3.5–6.0/90–110 ppm) (Figure 3), the H_1_/C_1_ signals of polysaccharides appeared. H_1_/C_1_ signals of cellulose (β-D-Glc*p*), xylan (β-D-Xyl*p*) and mannan (β-D-Man*p*) were observed at 4.46/102.9, 4.37/101.9 and 4.64/100.5 ppm, respectively. After molding, those volumes increased a lot, as in the citric acid type adhesive process [32]. The reducing end’s signals of mannan and xylan appeared at 5.04/94.2 and 4.39/97.7 ppm. Some of the hemicelluloses were degraded by the cleavages of the glycosidic linkages. In those cases, the reducing end’s glucose signals appeared at 5.07/92.5 (α) and 4.46/97.2 (β) ppm, too. However, it could be considered that these signals mainly contained the signals of glucose from the degradation of sucrose.

These results proved that the sucrose/citric acid adhesive could be defined as a chemical-bonding-type adhesive. This adhesive also functioned as a polymer produced from sucrose during the molding process as well as being shown in the previous section. It is considered that the adhesive mechanism in this case included both chemical bonding and secondary forces interaction. Thus, sucrose/citric acid type adhesive could be defined as a hybrid-type wood adhesive.

## 4. Conclusions

The structures of sucrose/citric acid type natural wood adhesive in the wood-based molding process were directly confirmed and clarified by HSQC-NMR analysis. During the adhesive process, various reactions proceeded in the two different fields, the adhesive and the interphase parts. In the adhesive part, sucrose was degraded under the acidic condition. In the presence of citric acid, the glycosidic linkage of sucrose was cleaved to produce glucose and fructose; then, these sugars converted to 5-HMF under high-temperature and high-pressure conditions with dehydration mechanisms. Ultimately, furan-type polymers were produced although its polymer structure could not be clarified in detail with the HSQC-NMR analysis. Moreover, the polymers were partly esterified with citric acid at the hydroxyl group of the 5-HMF-type structure. Then, in the interphase part, the esterification with citric acid proceeded at the primary hydroxyl groups of the wood components, as with the 5-HMF adhesive part. Chemical bonds between the wood and the adhesive were confirmed. At the same time, surface parts of the hemicelluloses, such as mannan and xylan, were damaged. Citric acid took on various roles during the adhesive process, perhaps most importantly, functioning as a clamp between the obtained furan polymer and the wood powder. The results suggested that the adhesive effect consists of the chemical bonding interaction derived from the ester bonds as well as the secondary forces interaction between furan polymers and wood components. As a result, we confirmed that the sucrose/citric acid type adhesive could be defined as a hybrid-type (both chemical bonding and secondary forces) wood adhesive.
